# Tinnitus services in the United Kingdom: a survey of patient experiences

**DOI:** 10.1186/s12913-018-2914-3

**Published:** 2018-02-13

**Authors:** Don McFerran, Derek J. Hoare, Simon Carr, Jaydip Ray, David Stockdale

**Affiliations:** 1 Colchester Hospital University NHS Foundation Trust, Department of Otolaryngology, Essex County Hospital, Lexden Rd, Colchester, Essex CO3 3NB UK; 2 NIHR Nottingham Hearing Biomedical Research Centre, Otology and Hearing Group, Division of Clinical Neuroscience, University of Nottingham, Nottingham, NH1 5DU UK; 3 Regional Department of Neurotology, Department of Otolaryngology, Royal Hallamshire Hospital, Sheffield, S10 2JF UK; 4 British Tinnitus Association, Ground Floor, Unit 5, Acorn Business Park, Woodseats Close, Sheffield, S8 0TB UK

**Keywords:** Tinnitus, Primary care, Secondary care, Revolving door, Audiology, Psychology

## Abstract

**Background:**

Tinnitus service provision in the United Kingdom has been investigated from the healthcare provider’s perspective demonstrating considerable regional variation particularly regarding availability of psychological treatments. An audiological-based tinnitus service, however, was reportedly available for all tinnitus patients in the UK. The aim of the current study was to define and evaluate nationwide tinnitus healthcare services from the patients’ viewpoint.

**Methods:**

Secondary analyses were performed on data from a 33-item questionnaire provided by the British Tinnitus Association. The questionnaire had been distributed via email and social media.

**Results:**

Responses from 937 participants who had or had previously experienced tinnitus were analysed. All but one person had at some time consulted their GP. About one in five received medication in primary care. The majority were referred to secondary care, generally an ENT surgeon or audiovestibular physician; some were referred directly to audiological services. In secondary care the majority underwent audiometric testing and over half underwent MRI scanning. Drugs were prescribed less frequently in secondary care. About one third of patients were referred onwards from diagnostic services in secondary care to receive therapeutic interventions for tinnitus. Therapy was generally delivered by an audiologist or hearing therapist. Just under two fifths of people discharged from secondary care returned to their GP, with most returning within one year. Over a third of this group were re-referred to secondary care. Few patients saw a psychologist (2.6%) though some psychological treatments were delivered by appropriately trained audiologists. Negative counselling from healthcare professionals in both primary and secondary care settings was reported.

**Conclusions:**

Although the UK has developed a national service for patients with tinnitus many people find it difficult to access, being blocked at the primary care level or after secondary care diagnostic services. Many of those discharged from secondary care return to their GP within a short space of time and are re-referred to secondary care creating an unsatisfactory and expensive revolving-door pattern of healthcare. Despite psychological treatment modalities having the best evidence base for successful tinnitus management, only a minority of tinnitus patients ever get to meet a psychologist.

**Electronic supplementary material:**

The online version of this article (10.1186/s12913-018-2914-3) contains supplementary material, which is available to authorized users.

## Background

Tinnitus, defined as the perception of sound in the absence of the corresponding external sound source is a common symptom: epidemiological studies from several countries have shown point prevalence figures generally between 10 and 15% of the adult population [1]. In the UK, the largest survey of tinnitus prevalence was undertaken as part of the National Study of Hearing in England, surveying 48,313 people [2]. Tinnitus in this study was reported by 10.1% with 2.8% describing their symptom as being at least moderately annoying. For 0.5% of respondents their tinnitus was having a severe effect on the ability to undertake activities of daily living.

Although a cure for tinnitus remains elusive there are many strategies for helping to ameliorate the impact of the condition [3, 4] including explanation and reassurance, counselling, management of associated hearing loss, sound therapy and psychological modalities such as cognitive behavioural therapy (CBT) [5–7], mindfulness meditation [8] and acceptance and commitment therapy (ACT) [9].

There have been various attempts at producing a set of structured guidelines for the management of tinnitus: The Department of Health in England issued a consensus document that presented commissioning guidelines and best practice advice in 2009 [10]; the American Academy of Otolaryngology published evidence-based clinical guidelines in 2014 [11]. The National Institute for Health and Care Excellence (NICE) have published a Clinical Knowledge Summary regarding management of tinnitus [12] but have not, to date, produced a formal Quality Standard.

Several service evaluations have accounted UK National Health Service (NHS) tinnitus services from the perspective of general practitioners (GPs), audiologists or hearing therapists (in the UK, hearing therapists are health care professionals who specialise in the rehabilitation of patients with hearing loss, tinnitus and other otological symptoms, but generally do not become involved with audiological testing or fitting of hearing instruments) [13–15]. In those service evaluations, clinicians provided an overview of what they perceive that they offer to patients with tinnitus, what resources they have and use and the training they have undertaken. They also provided opinions on what make for a good tinnitus service and how effective they considered their care to be. Surveys of healthcare providers tell part of the story but do not necessarily define the services as perceived by the end user. A recent publication has evaluated the effectiveness of different tinnitus and hyperacusis therapies from an end user perspective in a single UK hospital [16]. They concluded the most effective components of interventions to be counselling, education, and CBT. The current study was a retrospective analysis of anonymised data collected by the British Tinnitus Association (BTA) [17] and was developed to account the patients’ experience and evaluate nationwide tinnitus healthcare services from the patients’ viewpoint.

## Methods

This study was a service evaluation involving retrospective analysis of anonymised data originally collected by the BTA from its members to inform a cost of tinnitus care study [18]. Data were analysed with the support and permission of the data controller (DS). This use of the data complies with the governance procedures of the charity. As this study was a retrospective service evaluation and used only highly anonymised data for the sole purpose of service evaluation, individual consent was not sought, and research ethics committee review was not required [19].

### Questionnaire development

A 33-item questionnaire was developed in an iterative process involving staff and members of the Professional Advisers’ Committee of the BTA (tinnitus advisers, clinicians, researchers). Questions were developed based on experience with previous surveys of audiological tinnitus services [14], previous membership surveys conducted by the BTA (unpublished) and issues that had arisen during an assessment of the economic impact of tinnitus [18]. The 33 items comprised 5 demographic questions and 28 tinnitus specific questions (12 multiple choice, 13 multiple choice with an ‘other’ option and 3 open ended) (see Additional file 1).

The questionnaire was uploaded to an online data collection and analysis service, Survey Monkey [20]. The design used a separate page for each online question. No questions were mandatory. Participants were asked only to respond if they were resident in the UK, had, or previously had, tinnitus and had consulted their GP about tinnitus.

### Distribution

The questionnaire went live on Survey Monkey on 14^th^ of October 2014 and remained open until 31^st^ of October 2014. A link to the questionnaire was emailed to 4446 email addresses on the British Tinnitus Association (BTA) database. These were BTA members, people who had sought advice from the BTA, or recent BTA website users. The link to the questionnaire was also sent out using social media to people following the BTA via Facebook or Twitter. There was no paper version of the questionnaire. No explicit age restriction was created.

### Data management

Data were exported from Survey Monkey and managed in Excel. Two authors (DM and DJH) independently assessed the results to identify duplicate studies, those from outside the UK and any anomalous responses. Because of the anonymous nature of the study, it was not possible to ask respondents about missing data. It was assumed that questions were left blank when the respondent had no personal experience of that item or did not want to provide a response. DS was the data guarantor.

### Analyses

Quantitative analyses were performed in Excel. Open-ended questions were analysed by developing coding categories for each type of response and assigning codes to individual responses within the Excel spreadsheet. The codes were generated by one author (DM) and independently verified by another (DJH). The codes were then collated and analysed. Responses to open ended questions were used where appropriate to provide illustrative quotes that supported or helped explain quantitative findings.

## Results

A total of 977 responses were received. Twenty of these were identified as duplicates where participants are assumed to have started the questionnaire, paused and then re-started afresh. Twenty responses were identified as coming from outside the UK, despite the instructions stating that the survey was intended for people in the UK. These 40 entries were discounted from the analysis. A further entry was from a respondent living on the Isle of Man. The Isle of Man is a British Crown Dependency and as such is not officially a component part of the UK. However, Manx healthcare shares some services with the North West region of the UK and it was therefore retained in the analysis. In total, 937 responses were subjected to analysis.

Responses were received from all regions of the UK (Table 1), though comparing responses to population density figures from the Office for National Statistics [21] showed a slight overrepresentation from the South East (13.74% of UK population: 20.38% of survey respondents) and South West (8.40 of UK population: 13.34% of survey respondents).

**Table 1:** The regional distribution of responses to the questionnaire survey (*n* = 937)

Region	Respondents	
	(n)	(%)
England		
North East	36	3.84
North West	104	11.10
Yorkshire and The Humber	71	7.58
East Midlands	54	5.76
West Midlands	71	7.58
East of England	68	7.26
London	102	10.88
South East	191	20.38
South West	125	13.34
Wales	38	4.06
Scotland	67	7.15
Northern Ireland	9	0.96
Isle of Man	1	0.11

**Fig. 1:**
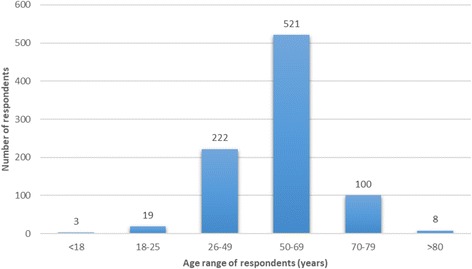
The age distribution of survey respondents (*n* = 873)

Eight hundred sixty-six respondents identified their gender: 440 women, 425 men and 1 transgender. Eight hundred seventy-three people identified their age (Fig. 1). The modal group by age distribution was the group aged between 50 and 69. This concurs with epidemiological studies of tinnitus which generally show that tinnitus is most prevalent in this age group [2].

### Primary care

**Fig. 2:**
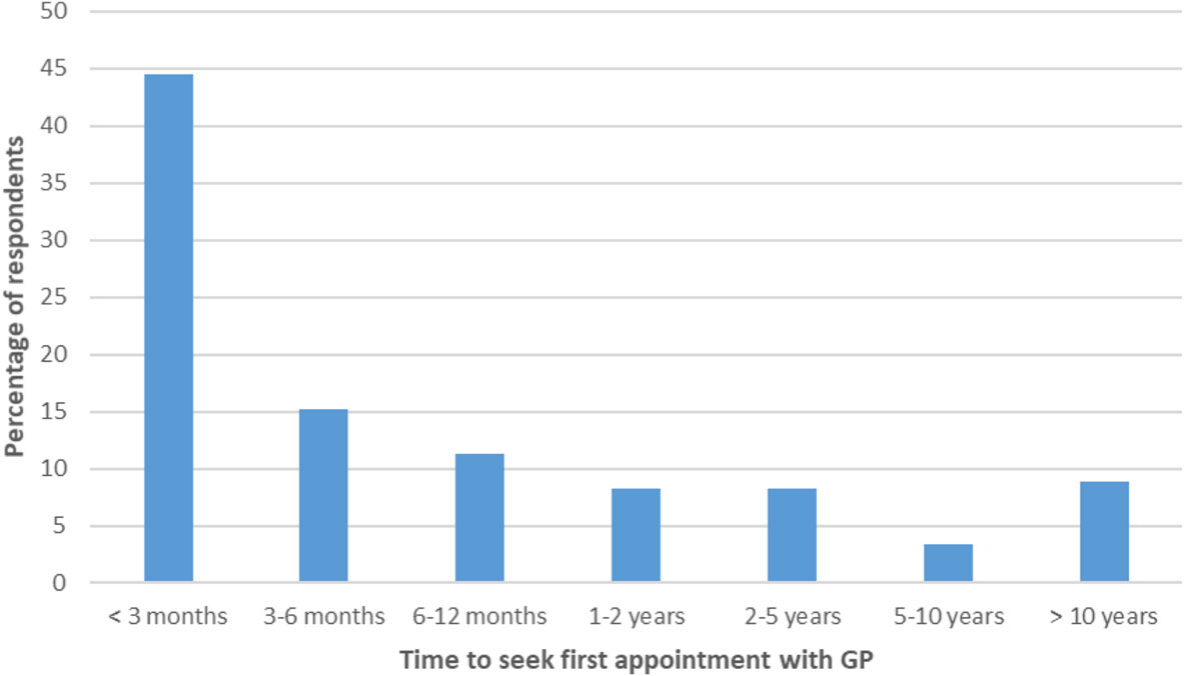
Time between onset of tinnitus and the patient first seeking an appointment with their GP (*n* = 931)

All but one respondent had seen their GP at some point regarding their tinnitus. Nine hundred thirty-one respondents answered the question “Approximately how long did you have tinnitus for before you saw your GP?” (Fig. 2). The majority (71.0%) made their first contact with their GP within 1 year of the onset of their tinnitus symptoms, with most of these (44.5% of the total) presenting within three months of onset.

Respondents reported that at their GP appointments a variety of interventions were offered (Table 2). The most common intervention was onward referral to secondary care (76.6%) after one or more GP appointments. Most frequently this referral was to the local ENT or Audiovestibular Medicine (AVM) service. Other common interventions included the supply of information (10.7%) or prescription of drugs (20.1%). When drugs were recommended, psychoactive drugs were the most common prescription, with 60 people reporting that they had been given an antidepressant, 26 had been given a hypnotic, and 2 had received an antipsychotic (Table 3). Nasal drugs were also commonly prescribed and seemed to be aimed at improving suspected Eustachian tube dysfunction. Twenty-four people received antibiotics. Twenty-five had been given betahistine but it is not clear whether this was because of suspected Ménière’s disease or as an off-licence treatment of subjective idiopathic tinnitus. Approximately one in five respondents (19.5%) reported that they did not receive any intervention from their GP. Negative statements made in response to open ended questions, such as “My GP didn’t seem bothered and he didn’t really want to explain anything to me, maybe he just didn’t know enough about the subject. He could of sent me to see a specialist who could help me further” were common.

**Table 2:** Interventions offered at the GP consultations. Percent figures are expressed as percentages of the respondents who had consulted their GP (*n* = 936)

Intervention	Respondents	
	(n)	(%)
Information provided	100	10.7
Referred to secondary care	718	76.6
Referred to ENT or Audiovestibular Medicine (AVM) physician	519	55.4
Referred to Audiology	267	28.5
Referred to ENT or AVM and Audiology	68	4.5
Medication prescribed	188	20.1
GP follow-up appointment arranged (watchful waiting)	32	3.4
None	183	19.5
Hearing test at the GP surgery	26	2.8
In-house dewaxing arranged	9	1.0
Referred to GP colleague with special interest in ENT (GPWSI)	10	1.1
Referred to high street service	2	0.2
MRI arranged	6	0.6

**Table 3:** Nature and number of drugs issued to tinnitus patients in primary (*n* = 931) and secondary care (*n* = 294)

Drug	Prescribed by GP (*n* = 931)		Prescribed by ENT/AVP (*n* = 294)	
	(n)	(%)	(n)	(%)
Antibiotics (systemic and topical)	24	2.58	1	0.34
Steroids	2	0.21	4	1.36
Betahistine	25	2.67	13	4.42
Vestibular sedatives	8	0.86	3	1.02
Nasal drugs	39	4.19	5	1.70
Cerumenolytics	3	0.32	0	0
Beta blockers	3	0.32	0	0
Analgesics and anti-inflammatories	4	0.43	0	0
Anticonvulsants	1	0.11	0	0
Psychoactive drugs				
Antidepressants	60	6.44	3	1.02
Hypnotics				
Benzodiazepines	9	0.97	0	0
Z-drugs	8	0.86	0	0
Unspecified hypnotic	9	0.97	1	0.34
Antipsychotics	2	0.21	0	0
Unsure of name of drug	15	1.61	2	0.68

**Fig. 3:**
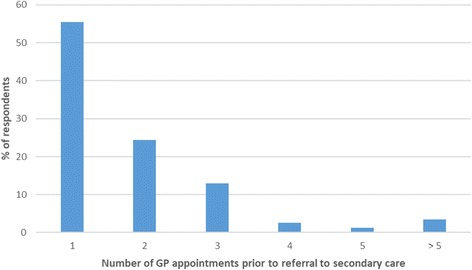
The number of visits to the GP prior to referral to secondary care (*n* = 680)

Six-hundred and eighty participants answered the question “If you were referred to hospital (ENT/Audiovestibular Medicine, or Audiology department), approximately how many times did you see your GP before you were referred?”. Although just over half of respondents (55.4%) reported that they were referred at their first visit and 79.8% had been referred by their second GP appointment, 20.2% attended on three or more occasions before a referral was made (Fig. 3). Twenty-one people stated that they had to demand or formally complain before their GP would consider referral to secondary care. Typical free text entries from this group included “Been at varying times to GP over the years with tinnitus, some GPs do nothing, some try medication, had to fight to get ENT referral” or “I was frustrated at the lack of support from the GP and had to push for a referral to ENT”. Five reported that they were still waiting for their secondary care appointments.

### Secondary care

Pathways were defined for 673 respondents (Table 4). Referral to a hospital based ENT/AVM service or hospital-based Audiology service was most common. Although they were not asked directly, 32 respondents stated within responses to open-ended questions that they had gone down a private healthcare route, either because they had pre-existing private healthcare insurance, because they felt this was the only way of obtaining a timely referral, or because they felt this was the only way of obtaining a referral from their GP at all. It is likely that the number of people who did use private healthcare was underestimated here as it was not specifically included in any question’s response option. Sixteen respondents identified their first point of contact with secondary care as being the radiology department for an MRI scan. It is likely that these patients had additional appointments with ENT or AVM.

**Table 4:** Initial referral pathways following Primary Care assessment, *n* = 673

Initial referral from GP	Respondents	
	(n)	(%)
Hospital based ENT or Audiovestibular Medicine	317	47.10
Hospital based Audiology	252	37.44
Community Audiology	13	1.93
Audiology within retail premises	3	0.45
Hearing Therapy	68	10.10
Armed Forces clinic	1	0.15
GP with Special Interest	1	0.15
Tinnitus support group	1	0.15
Mental Health services	1	0.15
Radiology department	16	2.38

Of the patients attending ENT/AVM, 294 described the diagnostic and therapeutic procedures that occurred at the first appointment or were booked from that appointment (Table 5). The majority (90.1%) reported having an audiometric assessment. Over half (59.2%) were referred for an MRI scan. Haematological testing was seldom requested (3.1%), as was referral for CT scanning or ultrasound scanning (2.1%). The proportion of patients prescribed drugs in secondary care was approximately half that in primary care (10.1% vs 20.1% respectively) and the pattern of drugs prescription was very different (Table 3). In 12 of the 32 patients (37.5%) who received drugs in secondary care, the drug was betahistine. Psychoactive drugs were infrequently prescribed in secondary care, being given to 4 out of 32 patients (12.5% of those who received drugs). Similarly, nasal drugs and antibiotics were prescribed much less frequently in secondary care.

**Table 5:** Diagnostic and therapeutic activities undertaken at first ENT/Audiovestibular Medicine appointment (*n* = 294)

Action at first appointment	Respondents	
	(n)	(%)
Audiogram and/or tympanometry	265	90.1
MRI scan	174	59.2
CT scan	4	1.4
Ultrasound scan	2	0.7
Blood test	9	3.1
Vestibular function testing	2	0.7
Brainstem evoked response audiometry	1	0.4
Removal of wax	3	1.0
Insertion of ventilation tube	1	0.4
Prescription of drugs	32	11.9

**Fig. 4:**
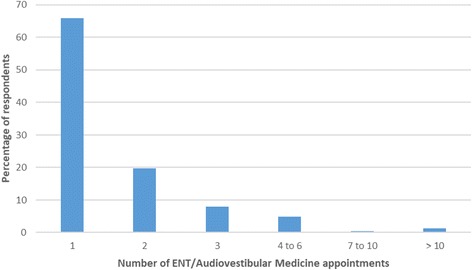
The number of ENT/Audiovestibular Medicine appointments attended per respondent (*n* = 249)

Two-hundred and forty-nine people identified the number of ENT/AVM appointments they had attended (Fig. 4). The majority (65.9%) had a single appointment and only 6.4% had more than 3 appointments.

Ultimately, most people with tinnitus underwent audiometry through a variety of different pathways (Table 6). Seven-hundred and ten people responded to this question and 696 (98.0%) reported that they had undergone a hearing test. Four had not yet been tested but had been given an appointment time for testing. Ten people reported that they had never had a hearing test.

**Table 6:** The location where patients underwent audiological testing

Location of audiometry	Respondents	
	(n)	(%)
GP surgery	26	3.74
Audiology within retail premises	15	2.16
Private medical practice	38	5.46
Occupational audiology service	1	0.14
NHS Audiology service	318	45.69
NHS Audiology at NHS ENT/AVM appointment	298	42.82

**Fig. 5:**
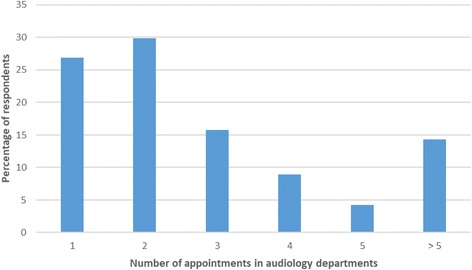
The number of audiology department appointments attended per respondent (*n* = 405)

**Fig. 6:**
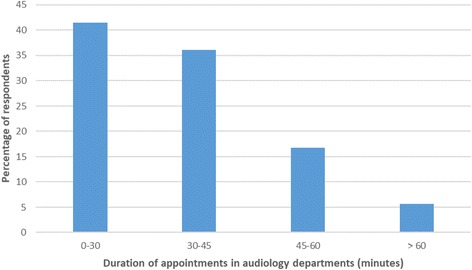
The duration of audiology department therapeutic appointments (*n* = 405)

After ENT/AVM assessment 67.7% were discharged and 32.3% of people were referred onwards to other secondary care services. The respondents who were referred onwards were generally seen in audiology departments for audiological management of their tinnitus. Some people reached audiology services by direct referral from their GPs. Overall, 273 respondents reported being seen by an audiologist and 114 by a hearing therapist. Forty-seven people were seen in audiology but were unsure whether they were under the care of an audiologist or hearing therapist. The number of appointments per respondent (Fig. 5) and the duration of these appointments (Fig. 6) varied.

Two hundred seventy respondents described the tinnitus services they had received in audiology departments (Table 7). Although treatment modalities such as CBT and mindfulness meditation are typically associated with psychological services, following pioneering work by Sweetow [22] a number of audiologists and hearing therapists have trained to offer such therapies within an audiological setting. Many people reported receiving multiple treatment modalities, with a mean of 1.8 techniques per person.

**Table 7:** Services offered in audiology departments for the management of tinnitus (*n* = 270). Some respondents received more than one service

Audiology department tinnitus management services	Respondents	
	(n)	(%)
Written information	180	66.67
Sound therapy plus education	95	35.19
Listening strategies	77	28.52
Relaxation	62	22.96
Cognitive behavioural therapy	29	10.74
Mindfulness meditation	24	8.89
Group education	19	7.04

Audiology departments also supply or demonstrate devices/equipment used in the management of tinnitus (Table 8). The hospital supplied equipment in 227 instances, and issued on loan in a further 24 cases. Thirty respondents reported that they bought their own equipment. Some hospitals supplied a limited range of devices/equipment such as sound generators free of charge but other items such as pillow speakers had to be purchased by patients.

**Table 8:** Devices supplied by audiology departments or demonstrated to patients for subsequent purchase (*n* = 270)

Devices supplied or demonstrated within audiology departments	Respondents	
	(n)	(%)
One hearing aid	65	24.07
Two hearing aids	93	34.44
White noise generators	91	33.70
Combination hearing aid device - hearing aid with sound generator	29	10.74
Sound therapy device e.g. table top sound generator	44	16.30
Pillow speakers	37	13.70
Relaxation CDs	34	12.59

After a course of audiological management, 69.3% of respondents were discharged back to primary care or to self-manage whereas 30.7% were given an open appointment to facilitate return to audiological services if necessary.

Only 24 respondents reported that they had seen a psychologist regarding their tinnitus. Of these, 7 had received a course of CBT, 3 had received mindfulness meditation, and 4 had received both modalities. The other 10 respondents who had received a psychological intervention did not specify the approach used.

**Fig. 7:**
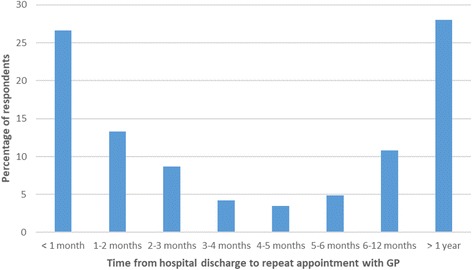
The time taken from discharge from hospital to seeking a repeat appointment with the GP (*n* = 286)

Eight hundred sixty-eight people answered the question “Have you been back to your GP regarding your tinnitus?” after discharge. Of these, 334 (38.5%) answered in the affirmative. Two hundred eighty-six respondents were able to define the time that elapsed from discharge from hospital to return to GP (Fig. 7). 72.0% had returned within a year and for 39.9% of the sample, return was in less than 2 months. Of the 334 people who went back to their GP, 122 (36.5%) were referred back to secondary care.

The final tinnitus -related question in the survey invited respondents to supply any additional information that they felt would be of use. Six hundred eight people responded to this question, supplying a total of 852 separate observations. One hundred twelve respondents simply described the nature of their tinnitus. The remainder made a wide variety of statements regarding the helpfulness or otherwise of facets of tinnitus service in the UK (Table 9) or made statements regarding deficiencies in the service with suggestions for improvement (Table 10).

**Table 9:** Personnel and management strategies that people found helpful or unhelpful in their tinnitus journey (*n* = 608)

	Helpful		Unhelpful	
	(n)	(%)	(n)	(%)
People and departments				
The healthcare system generally	5	0.82	19	3.13
General Practitioners	28	4.61	102	16.78
Hospital based services	8	1.32	12	1.97
ENT Surgeons and Audiovestibular Physicians	14	2.30	30	4.93
Audiologists and Hearing Therapists	36	5.92	15	2.47
Nurses			2	0.33
Community Audiology services			1	0.16
High Street Audiology services			4	0.66
Tinnitus support groups	10	1.64		
Another person with tinnitus	2	0.33		
Tinnitus management				
Hearing aids	31	5.10	7	1.15
Sound therapy devices	14	2.30	3	0.99
Psychological treatments	2	0.33	4	0.66
Medication (hypnotics or antidepressants)	3	0.49	1	0.16
Having open access to support services	6	0.99		
Sources of information				
Charities	23	3.78		
Internet	9	1.48	1	0.16

**Table 10:** Observations regarding tinnitus services, including unhelpful attitudes and suggestions for improvements (*n* = 604)

	Respondents	
	(n)	(%)
Comments regarding tinnitus services		
Services poor or unavailable locally: had to travel	12	1.99
Paid for private treatment	32	5.23
Had to go through the system more than once: “revolving door” healthcare	8	1.32
Had to be very persistent to obtain referral to tinnitus services	21	3.48
Learnt to self-manage tinnitus	12	1.99
Gave up interacting with healthcare services	2	0.33
Factors that would improve tinnitus services		
More information	11	1.82
A quicker service from tinnitus services	42	6.95
More extensive diagnostic services	15	2.48
Easier booking of follow-up appointments	8	1.32
Easier access to audiology	2	0.33
Easier access to psychology	6	0.99
More general recognition and awareness	31	5.13
More knowledge of local tinnitus options by General Practitioners	9	1.49
More research and /or a cure	19	3.15
Negative experience of tinnitus services		
Told by healthcare professional that nothing can be done/they will have to live with the tinnitus	201	33.28
Patient concluded that nothing can be done/they will have to live with the tinnitus	19	3.15

## Discussion

This is the first account of the patient experience of tinnitus services in the UK generated from a large patient survey.

Nearly half of respondents (44.5%) to the survey presented to their GP within three months of the onset of their tinnitus and almost three quarters had done so by the one year mark. Most respondents obtained onward referral to ENT/AVM or audiology which is similar to the findings of a survey of audiology departments [15] though only 55.4% were referred after their initial consultation. Among those patients who were not referred immediately, initial management typically consisted of information provision, prescription of medication or watchful waiting. Surprisingly, almost one fifth of the respondents (19.5%) stated that their GP took no action whatsoever at their first appointment. It is interesting to contrast patient perceptions of management with those of GPs: A survey of UK GPs [13] found that 35% of GPs reported that they gave out information leaflets regarding tinnitus. When patients were asked a similar question in the current survey only 10.7% reported receiving information. When GPs did provide tinnitus information in the current study, they did so either directly or by referring patients to internet resources. Furthermore, a survey of the online information sites favoured by GPs has shown considerable variation in the quality of information that is offered [23].

There were some notable differences in the prescribing practices of primary care and secondary care physicians. The figures regarding drug prescription in primary care (20.1%) tallies closely with the estimate of 17% reported in the GP survey [13]. Common prescriptions were psychoactive drugs, antibiotics, both systemic and topical, betahistine or nasal drugs such as decongestants and topical steroid sprays. In secondary care, only 10.1% received drugs and the common prescriptions were betahistine, nasal drugs and systemic steroids. The difference in overall number of prescriptions was significant (Fisher exact test *p* < 0.0001) and there was also significant difference in some of the types of prescribed drug with antibiotics, nasal drugs and antidepressant drugs more likely to be prescribed in primary care (Fisher exact test, *p* = 0.0159, *p* = 0.0478 and *p* < 0.0001 respectively). By contrast, systemic steroids were more likely to be prescribed in secondary care (Fisher exact test *p* = 0.0322). Antibiotics are clearly an appropriate management if the tinnitus has been triggered by a bacterial infection. It is, however, notable that only one respondent reported receiving antibiotics from a secondary care physician. Tinnitus is not infrequently accompanied by a blocked sensation in the ear. It seems likely that physicians without access to advanced diagnostic equipment interpret this as evidence of infection and prescribe antibiotics accordingly. Similarly, this blocked sensation may be misinterpreted as evidence of Eustachian tube dysfunction. Nasal drugs to improve Eustachian tube function are then the logical treatment. There were almost 8 times more nasal drugs prescribed in primary care compared to secondary care, again suggesting that once diagnostic equipment such as tympanometers or operating microscopes were available to clinicians a more accurate diagnosis could be made. There is no robust evidence to support the use of betahistine in the management of spontaneous idiopathic tinnitus [24], but this drug may be appropriate if tinnitus is a component of Ménière’s disease. In their clinical practice guidelines, Tunkel et al. [11] recommended against the use of antidepressants, anxiolytics, anticonvulsants and intra-tympanic medication for the routine treatment of persistent, bothersome tinnitus. Psychoactive drugs do, however, have a role when there is comorbid psychological illness, generally anxiety or depression [25]. The large difference in the prescription of psychoactive drugs between primary and secondary care probably reflects that ENT physicians do not feel that it is part of their remit to manage mental health conditions. Some respondents reported that they had been prescribed amitriptyline, specifically to access what is usually regarded as a side effect, namely its sedative properties.

Clinical guidelines and best practice documents recommend that all patients with tinnitus should undergo a basic audiometric assessment and ultimately the majority of respondents who answered this section of the questionnaire did undergo hearing tests. The process by which this happened appeared haphazard with multiple different pathways and no standardisation of when the testing should occur. A small number of respondents (1.1%) reported that they had never undergone a hearing test, which does seem to be a shortcoming in their care.

After audiological testing, the only diagnostic test that was performed regularly was MRI scanning (59% of those assessed by ENT or AVM underwent an MRI scan). The most common reasons for requesting medical imaging are unilateral or significantly asymmetric tinnitus or asymmetric audiometric tests [26]. As epidemiological studies have shown that approximately half of people with tinnitus have unilateral tinnitus [27], this rate of investigation seems appropriate. Use of such diagnostic tests carries financial implications which are discussed in a separate study [18].

It is perhaps surprising that only about a third of patients who underwent diagnostic assessment by ENT/AVM were subsequently referred onwards for therapeutic management of their symptom. The survey made no attempt at determining the severity of individual respondents’ tinnitus. It is therefore possible that some people simply wanted the reassurance of knowing there was no serious underlying pathological reason for their tinnitus and were then content to self-manage. Although figures did not reach statistical significance, a higher proportion of patients who were discharged after investigation returned to their GPs compared to those who were referred for audiological tinnitus management.

It is illuminating that 38.5% of patients re-presented to their GP regarding their tinnitus, and for 39.9% of these, return was within the first two months after discharge from the hospital. Of those returning to primary care, 36.5% were referred back to hospital, 24% were prescribed medication and 39% were offered no further help. Thus, one in eight of the overall survey population went through secondary care, returned to primary care and were then re-referred to secondary care. Although this ‘revolving door’ pattern is well recognised in various healthcare areas such as mental health services [28] and care of the elderly [29], this is the first time the phenomenon has been reported by tinnitus patients. The survey did not explicitly ask why people went through the process two or more times, though several reasons are possible: it may be that the treatments offered were ineffective, or their tinnitus changed or that they were dissatisfied with the service they had received. A survey of GPs and ENT specialists in Western Europe, UK and USA [30] found a low success rate of tinnitus therapy. Whatever the cause, revolving door healthcare is time consuming for both patient and health care professional and carries significant financial cost implications [18].

The evidence base for the efficacy of the available tinnitus management strategies is limited but there is there is a reasonable level of scientific evidence of the effectiveness of psychological therapies particularly cognitive behaviour therapy in the management of tinnitus [3, 7]. It was therefore surprising to see that the people who commented on psychological treatment of tinnitus were more likely to describe it is as unhelpful by a factor of 2.0. Admittedly this was a very small sample (*n* = 6) and it would be wrong to read too much into the observation. However, only 24 respondents (2.6%) reported that they had seen a psychologist. Psychological treatments were also delivered within some audiology departments by audiologists and hearing therapists. It is not standardised however and there is currently no research evidence for the effectiveness of an audiologist or hearing therapist psychological intervention [31].

Free text responses to the final tinnitus-related question in the survey give considerable insight into patients’ perceptions of tinnitus services. Physicians within the tinnitus pathway were considerably more likely to be seen as unhelpful rather than helpful. One sixth of the people who replied to this question (102 of 604) were critical of their GPs management. By contrast audiologists and hearing therapists were more likely to be described as helpful, by a factor of 2.4. There are models of tinnitus care in the United Kingdom in which audiologists see tinnitus patients via direct referral pathways and undertake the majority of the care process: this is an area that merits further investigation.

Sound therapies were generally seen as helpful. This is particularly interesting as both sound therapies and hearing aids are widely used in tinnitus clinics [32], the latter for even quite modest hearing losses, but there is scant scientific evidence regarding efficacy [33–36].

The most common factor that people would like to see improved was the speed of delivery of tinnitus services: 7.0% of people who answered this question felt that they had experienced unacceptable delay. Although this was seen as a problem within GP and ENT services, other issues may be involved. Clinical Commissioning Groups in some cases stipulate that patients with tinnitus should not be immediately referred to secondary care. For example, the guidelines produced by North East Essex Clinical Commissioning Group [37] recommend referral only if there is (1) consistent bilateral tinnitus (persistent for over 20 weeks) and hearing loss, or (2) unilateral tinnitus (persistent over 2 months). Exploring other forms of tinnitus intervention may help to deliver a more timely service. For example, internet delivered CBT has been successfully trialled in other countries [38]. A pilot study has been undertaken in the UK and more rigorous research is underway [39–42]. The effectiveness and cost effectiveness of these alternative pathways need to be investigated.

### Strengths and weaknesses

Strengths of this study include a large sample size and the fact that the survey achieved good representation from all health regions of the UK. Furthermore, the age and gender demographics of the survey population are broadly congruent with those seen in epidemiological studies of tinnitus. The use of Internet-based questionnaires is a contentious topic in healthcare research. Internet-based questionnaires offer several advantages over other data collection methods – distributing the survey is quicker and cheaper than paper, telephone and face-to-face surveys. Completing Internet surveys is quicker for participants than a telephone interview. There are however, some limitations of Internet questionnaires that may introduce bias [42, 43]. The response rate of Internet-based questionnaires is generally lower than with other forms of data collection and, in particular, there is a tendency for participants in Internet based studies not to complete all sections of the survey. Poor overall response rates can cause selection bias. Failure to answer all sections can generate response bias. A potential source of sampling bias is the makeup of the database that was used to contact participants. People interacting with the BTA may have more intrusive tinnitus than average or may be interacting with the charity because they are dissatisfied with the service that their healthcare providers have supplied. The age of participants may also be relevant. Government statistics show that 89% of the UK population interact regularly with the Internet but this varies from 99% in the 16-34 year old age range to 41% in those over 75 [44]. This means that any internet-based questionnaire is likely to target younger participants disproportionately.

The study was retrospective and reliant on people’s memories. Some had experienced tinnitus for a considerable length of time, with 11.95% reporting that it was more than 10 years since they had first consulted their GP. This may introduce recall bias.

A further limitation might be the restrictiveness of some of the survey response options: a limited number of choices was offered without an ‘other’ category on some questions. The survey may have missed capturing data on components such as ‘self-management’ or on uncommonly used treatment possibilities. This can generate response bias. However, the survey did not insist that every question was answered and it was not a forced choice questionnaire. Free text entry was utilised at various points and participants were encouraged to include any comments that they thought might be relevant.

## Conclusions

In comparison with most other countries, the UK has well developed tinnitus services with a network of audiology departments offering a range of tinnitus management paradigms and in England’s case a national best practice document. Asking the end user, however, tells a different story: one fifth of patients reported that at initial consultation their GP did nothing; two thirds of people who are referred for diagnostic services in secondary care are then discharged without any therapeutic intervention; one in eight people with tinnitus are referred from primary to secondary care, discharged and then re-referred to secondary care, creating expensive and unsatisfactory revolving door healthcare; only one in forty are able to access psychological services for tinnitus, despite an evidence base that suggests psychological treatments are the most efficacious management strategies. One third of patients report nihilistic attitudes from healthcare professionals. Clearly change is needed: tinnitus management at primary care, counselling/CBT in secondary care, and alternative referral pathways are priorities for both research and service development.

## Additional file

Additional file 1: Survey questionnaire: A tinnitus patient’s journey. The online questionnaire that was circulated to participants in the study. (DOCX 15 kb)

## Abbreviations

ACT: Acceptance and Commitment Therapy
AVM: Audiovestibular Medicine
BTA: British Tinnitus Association
CBT: cognitive behavioural therapy
CT: Computed Tomography
ENT: Ear Nose and Throat
GP: General Practitioner
MRI: Magnetic resonance imaging
NICE: National Institute for Health and Care Excellence
