# Supplementary material for: Tinnitus services in the United Kingdom: a survey of patient experiences
Source: BMC Health Serv Res. 2018 Feb 13;18:110. doi: 10.1186/s12913-018-2914-3 (PMC5809968; doi:10.1186/s12913-018-2914-3)
Supplement: Supplementary file 1 — Survey questionnaire: A tinnitus patient’s journey. The online questionnaire that was circulated to participants in the study. (DOCX 15 kb) [file 12913_2018_2914_MOESM1_ESM.docx]

**Survey questionnaire: A tinnitus patient's journey**

This survey is intended for people in the UK who currently have or have had tinnitus and have been to their GP about it. Please take some time to complete the survey, the details you give are very important to us.

If you have never experienced tinnitus, we thank you for your interest, but please do not complete this survey.

If you have any questions or comments, please feel free to contact the British Tinnitus Association

1. Approximately how long did you have tinnitus for before you saw your GP?
2. Approximately how long ago did you have your first appointment with a GP, about your tinnitus?
3. When you went to your GP about tinnitus what happened:
4. If your GP prescribed you medication, please tell us what it was:
5. How long did you take this medication?
6. If you were referred to hospital (ENT/Audiovestibular medicine, or Audiology department), approximately how many times did you see your GP before you were referred?
7. If you had a hearing test, where was this done?
8. If you were referred onwards, where did you get seen?
9. If you were referred to ENT/Audiovestibular medicine, what happened at this appointment?
10. Were you prescribed any medications by ENT/Audiovestibular medicine?
11. Did you have more than one appointment at ENT/Audiovestibular medicine?
12. Were you referred onwards from ENT/Audiovestibular medicine?
13. Were you seen in the Audiology department?
14. Who did you see in the Audiology department?
15. How many appointments did you have at the Audiology department?
16. Are you still being seen at Audiology?
17. Approximately how long were your Audiology appointments:
18. Within Audiology or a tinnitus clinic, have you had any of the following:

- Sound therapy plus education
- Group Educational sessions
- CBT (Cognitive behaviour therapy)
- Mindfulness
- Hearing Therapy listening strategies
- Relaxation
- Written information

1. Were you given any equipment to help you manage tinnitus?
2. If yes, please tell us what:

- One Hearing aid
- Two hearing aids
- White noise generators
- Combination hearing aid device - hearing aid with sound generator
- Sound therapy device e.g. table top sound generator
- Pillow speakers
- Relaxation CD/s

1. Please tell us who paid for the device/s:
2. Did you see a psychologist?
3. Have you now been discharged from Audiology?
4. Have you been given an open appointment by Audiology if you need further support for your tinnitus?
5. Have you been back to your GP regarding your tinnitus?
6. If you have been back to see your GP about your tinnitus, how long was it after being seen at the hospital or high street service, that you went back?
7. What happened when you went back to your GP?

- I was re-referred to the hospital
- I was given a hearing test
- I was prescribed medication
- Nothing

1. Please tell us anything else you feel would be of use:
